# HPV vaccine uptake among adolescent girls in Nigeria: The complex role of caregivers’ education

**DOI:** 10.1371/journal.pone.0325684

**Published:** 2025-07-08

**Authors:** Sohail Agha, Ifeanyi Nsofor

**Affiliations:** 1 Department of Epidemiology, University of Washington, Seattle, Washington, United States of America; 2 Behavioral Insights Lab, Seattle, Washington, United Stats of America; 3 Family Health Initiative, Abuja, Nigeria; Federal University Otuoke, NIGERIA

## Abstract

**Introduction:**

Cervical cancer remains a leading cause of cancer-related deaths among women in low- and middle-income countries (LMICs), with sub-Saharan Africa (SSA) bearing a disproportionate burden of the disease. Human papillomavirus (HPV) vaccination offers a critical intervention, yet uptake remains suboptimal due to vaccine hesitancy, misinformation, and socio-economic disparities. This study examines factors associated with HPV vaccine uptake among adolescent girls whose caregivers use social media.

**Methods:**

We conducted a cross-sectional survey in October and November 2024 among 4,830 caregivers of adolescent girls 9–17 in Abuja, Nasarawa, and Adamawa states. Participants were recruited via advertisements on Facebook and Instagram. Data on adolescents’ HPV vaccination was collected from caregivers. Caregiver also provided data on their own education, motivation, ability, and exposure to HPV vaccine messaging. Multivariate logistic regression was used to identify predictors of vaccine uptake, adjusting for socio-demographic factors, motivation, and ability.

**Results:**

The HPV vaccination rate among adolescent girls 9–17 was 53.9%. Caregivers with no formal education had higher exposure to HPV campaign messaging than caregivers with Higher National Diploma (HND) or Bachelor’s (BSc) education (95.3% vs 53.8%, p < 0.001). The least educated caregivers were also more likely to report a three times higher odds ratio of HPV vaccination compared to caregivers with Higher National Diploma (adjusted odds ratio [aOR] = 3.01, 95% CI: 1.52–5.93). Exposure to HPV vaccine messaging was associated with a seven times higher odds ratio of HPV vaccine uptake (aOR = 6.87, 95% CI: 6.20–7.61). Motivation and ability were positively associated with HPV vaccination. Regional differences were observed, with Nasarawa demonstrating higher a vaccination rate than Abuja and Adamawa.

**Conclusion:**

Exposure to HPV vaccine messages is higher among less educated compared to more educated caregivers. Moreover, the impact of advertising exposure on vaccine uptake is stronger among less educated caregivers. Educational disparities in campaign exposure and campaign effects highlight the need for strategies to increase campaign reach to more educated caregivers and to ensure that HPV messages resonate with them. Our findings suggest that existing campaigns may need to be restructured to more effectively reach educated and skeptical audiences.

## Introduction

Cervical cancer remains one of the deadliest cancers among women in low- and middle-income countries (LMICs), disproportionately impacting women in Nigeria [1]. The introduction of HPV vaccination in national immunization programs offers a transformative solution, but vaccine uptake remains inconsistent, with socioeconomic disparities and the growing influence of misinformation shaping vaccination behaviors [2,3]. This study leverages insights from 4,830 caregivers of adolescent girls recruited via social media platforms, to explore how a Nigerian caregiver’s education, gender, motivation and ability shapes their decision to vaccinate their child.

While formal education is generally associated with informed health decisions and preventive behaviors, recent evidence reveals a surprising level of complexity in its relationship with HPV vaccination in SSA. Studies in Nigeria and Kenya reveal a paradox: more educated caregivers report both lower intentions to vaccinate and reduced HPV vaccination rates among adolescents under their care [4,5]. This may reflect greater critical evaluation of vaccination campaigns or greater exposure to conflicting information among caregivers with higher formal education.

Studies have highlighted how social media influences vaccination behaviors, amplifying both positive and negative perceptions depending on social cues and how messages are framed [6]. The perceived credibility of sources of health information may also vary by level of education [6]. Persons with formal education may also assess the quality of social media messages differently from others [6]. Thus, a variety of factors may explain why caregivers with higher education levels may exhibit lower vaccine uptake in certain contexts.

The surge in HPV vaccine rejection rates in Nigeria underscores the complexity of these dynamics. Despite the vaccine’s proven safety and efficacy, misinformation and limited awareness of its benefits continue to undermine public confidence [5,7]. For instance, rumors of the HPV vaccine’s association with infertility and promiscuity are widespread in Nigeria [8]. To address barriers to HPV vaccine adoption, multiple strategies have been recommended, including evidence-based interventions that integrate HPV vaccine education into school curricula, digital media campaigns, and engaging religious and cultural leaders to dispel myths and promote vaccine acceptance [5,7].

Studies of social media users offer a unique lens to understand vaccination behaviors among digitally active populations. While digital media can propagate misinformation, it is increasingly being used as a critical channel for health promotion by global public health organizations such as UNICEF and GAVI to promote vaccination in LMICs [9].

This study draws on data from a survey conducted in Abuja, Nasarawa, and Adamawa states in Nigeria. The survey was conducted as a baseline for a social media campaign to promote HPV vaccination under the GAVI-funded VaxSocial program. The analysis provides valuable insights on the relationship between education and HPV vaccine uptake among caregivers of adolescent girls who use social media. The survey questionnaire is based on a behavior model that has been tested with multiple populations in Nigeria and other LMICs [4,9]. By examining factors associated with HPV vaccine uptake in Nigeria, this paper seeks to unravel the paradoxical role served by education in vaccine acceptance observed in certain contexts. The findings seek to inform targeted social media campaigns to address hesitancy experienced by more educated caregivers, with the objective of advancing equitable access to HPV vaccination and reducing cervical cancer disparities.

## Materials and methods

### Media campaign supporting HPV vaccination

In October 2023, Nigeria initiated a comprehensive communication strategy to promote Human Papillomavirus (HPV) vaccination, utilizing various media platforms to reach diverse audiences. Social media platforms played a pivotal role in disseminating information about the HPV vaccination campaign. The United Nations Children’s Fund (UNICEF) collaborated with public figures, actors, and social influencers to raise awareness about routine immunization, HPV vaccines, and cervical cancer prevention. These influencers engaged communities through posts and live sessions, emphasizing the vaccine’s safety and efficacy. UNICEF also produced informational materials, including radio and TV jingles in multiple local languages, to dispel misinformation and rumors about the HPV vaccine. These broadcasts aimed to educate the public on the importance of vaccinating adolescent girls aged 9–14 years and preventing cervical cancer. National newspapers covered the HPV vaccination initiative, highlighting the campaign’s progress and encouraging parents to vaccinate their daughters. The communications approach was broadly consistent with the approach used by UNICEF during Bangladesh’s HPV vaccinate introduction in 2023 [10].

### Study design

This study utilized a cross-sectional survey conducted by the Behavioral Insights Lab in October and November 2024. The survey was intended to serve as a baseline for a social media campaign to be initiated by Upswell in November 2024. It examines caregiver characteristics associated with adolescent girls’ HPV vaccination. The survey interviewed caregivers residing in three states in Northern Nigeria: Abuja, Nasarawa, and Adamawa. It allows us to identify factors associated with HPV vaccine uptake and offers valuable insights into the factors shaping HPV vaccine uptake within a year of its rollout.

### Sampling strategy

A sample size of 4,830 caregivers was achieved across the three states. Respondents were enrolled into the study through digital advertisements on Meta platforms (Facebook and Instagram). Eligible participants were caregivers of adolescent girls aged 9–17 years, particularly those aged 9–14. Recruitment efforts were designed to reach a cross-section of caregivers within the study regions. Advertisements to promote survey participation were tailored to appeal broadly across socioeconomic and demographic strata.

### Participant recruitment, data collection, and ethical approval

Participants were recruited via advertisements on Facebook and Instagram, targeted by geographic location, age, and gender to ensure demographic representation. Advertisements were designed to reach caregivers aged 18 and older in the study states and described the study purpose, including information about compensation of mobile credit (approximately USD 0.24) upon survey completion. Respondents who identified themselves as caregivers of adolescent girls aged 9–17 were eligible to participate.

Upon clicking the advertisement, respondents were directed to a web-based survey within the Facebook app. The survey began with an introductory message explaining the study purpose, emphasizing the voluntary nature of participation, and providing compensation details. Participants provided implied consent by clicking “start” to initiate the survey. To encourage participation, respondents could complete the survey at their convenience, with the option to pause and resume. The survey data collection was initiated on 12-10-2024 and was completed on 6-11-2024. Only fully completed surveys were included in the analysis. The digital recruitment campaign reached over 1.6 million individuals, generating 47,968 link clicks and resulting in 4,830 completed survey interviews. The survey was designed in clear, accessible language and delivered in both English and Hausa where applicable, the two most common languages spoken in the study areas.

The study protocol, including the consent process, was reviewed and approved by the National Health Research Ethics Committee of Nigeria (NHREC) on July 15, 2024, under protocol number NHREC/01/01/2007–15/07/2024.

### Inclusivity in Global Research

Additional information regarding the ethical, cultural, and scientific considerations specific to inclusivity in global research is included in the Supporting Information (S1 Checklist).

### Measures

#### Primary outcome.

The survey instrument used questions asked in previous surveys of HPV vaccination in Nigeria [4]. The primary outcome variable was HPV vaccination status. Caregivers were asked, “Has a girl-child in your care received an HPV vaccination?” Adolescent girls whose caregivers responded in the affirmative were classified as having been vaccinated. Responses were categorized as “Yes,” “No,” or “I don’t know”. Affirmative responses were considered as indicative of a child being vaccinated.

#### Motivation.

Motivation was measured using items informed by the Fogg Behavior Model (FBM), which emphasizes aspiration, sensation, and belonging as key elements within motivation that serve as drivers of behavior [11]. Caregivers rated their agreement with the statement, “Getting the girl who is in my care vaccinated against HPV is important to me,” on a five-point Likert scale ranging from “Strongly disagree” to “Strongly agree.” Caregivers who selected “Strongly agree” as the response were categorized as having a high level of motivation.

#### Ability.

To assess caregivers’ ability to get their child vaccinated, survey participants were asked, “To what extent do you agree with the statement: ‘I know where to get the HPV vaccine for the girl in my care?’” Caregivers who selected the “Strongly agree” response were classified as having high ability. Additional questions evaluated potential barriers faced by caregivers, including logistical challenges and difficulty accessing vaccination services. This survey item was also rated on a five-point Likert scale.

#### Sociodemographic characteristics.

The survey captured key sociodemographic variables, including caregiver gender, age, education level, and the age of the adolescent girl in their care. Education levels were categorized as no formal education, primary school, secondary school (SSCE/GCE), tertiary education (OND, HND, BSc), or postgraduate degree.

#### Exposure to campaign messaging.

Caregivers’ exposure to HPV vaccine-related messages was assessed by asking “Have you seen or heard any advertisements or messages about HPV vaccine in the last 3 months?” Responses were coded as “Yes” or “No” to measure caregivers’ exposure to the first year of the government’s vaccination campaign. The question on exposure refers to messages about the HPV vaccine from any source. Findings from a formative, research survey conducted by the Behavioral Insights Lab in Abuja, Nasarawa and Adamawa a few weeks earlier, in September 2024, showed that caregivers’ exposure to HPV vaccine messages was primarily through social media (52.8%). Exposure to vaccine messages from other sources was much lower, with television (11.3%), radio (11.3%), pharmacy (10.4%), primary health care center (8.5%) and billboards (1.9%) comprising the remaining sources of information about the vaccine [12].

### Statistical analysis

Survey data were analyzed using univariate, bivariate, and multivariate techniques. Univariate analyses examined frequency distributions of all variables to understand characteristics of the sample. Bivariate analysis explored associations between education, motivation, ability, and HPV vaccination status, with chi-square tests employed to determine statistical significance at p < 0.05.

Multivariate logistic regression was used to calculate adjusted odds ratios (aORs), identifying the relationships between education and HPV vaccine uptake while adjusting for potential confounders. Variables associated with the outcome in the bivariate analysis were included in the logistic regression model. Demographic factors, such as caregiver’s age, gender, state of residence, and the age of the adolescent girl in their care, were also controlled for. The analysis accounted for clustering within strata to ensure robust estimates. All statistical analyses were performed using STATA software (version 14).

We used data on all caregivers in our sample, even though the national guidelines for HPV vaccination relate to 9–14-year-olds. A relatively small proportion of caregivers in our sample (10.8%) were caregivers of girls ages 15–17. We conducted the analysis both with caregivers of girls ages 9–14 and with caregivers of girls ages 9–17 to ensure that the inclusion of 15–17-year-olds did not lead to any change in the inferences drawn from the analysis. Given the large overall sample size of the study (n = 4,830), there were no significant differences in the findings whether caregivers of 15–17-year-olds were included or excluded from the sample for the statistical analysis. It is noteworthy, however, that the rate of HPV vaccination was higher when the 15–17-year-olds were excluded from the analysis (57.2% versus 53.9%).

### Study limitations

There are several limitations inherent to its design and data collection approach:

1. **Selection Bias**: Recruitment through social media platforms likely excluded caregivers without access to social media accounts or the internet. As a result, the sample may over-represent digitally connected, younger, urban caregivers, limiting the generalizability of the findings.
2. **Self-Reported Data**: The reliance on self-reported vaccination status introduces the potential for recall or social desirability biases. Respondents may have over-reported or under-reported their child’s HPV vaccination status due to memory inaccuracies or their perceptions of the acceptability of HPV vaccination.
3. **Single Time Point Data Collection**: Cross-sectional survey data provides only a snapshot of caregiver attitudes, beliefs, and behaviors, precluding causal inferences. A longitudinal design is more likely to capture temporal changes and provide insights into how motivation, abilities, and vaccination behaviors evolve over time.
4. **Sample Composition**: Male caregivers were over-represented in the sample compared to female caregivers, an atypical finding for caregiver-focused studies. This disproportion may reflect that a higher proportion of men than women use social media in Nigeria. Alternatively, it may reflect biases in digital recruitment methodologies or underlying demographic differences in survey response rates among social media users.
5. **Social Desirability Bias**: While concerns about social desirability bias among less-educated respondents are valid, the broader literature suggests that socioeconomic status (SES) is only modestly associated with social desirability bias, and findings are often mixed. For example, some studies report higher social desirability bias among lower SES groups, particularly among women and older adults, but find minimal or non-significant effects after adjusting for education and employment. Other studies show that lower SES groups often demonstrate lower vaccine uptake. Possibly because of the mixed findings about the relationship between SES and vaccine hesitancy, the literature does not link socio-economic disparities in vaccination rates to systematic reporting biases [13–16]. We believe that social desirability bias is unlikely to account for the observed differentials in HPV vaccination uptake found in this study.

## Results

### Univariate analysis

The study sample comprises of 4,830 male and female caregivers aged 18 and older who have access to social media platforms such as Facebook and Instagram. Table 1, column 1, shows socio-demographic characteristics of caregivers in the sample and their motivation and ability. The digital survey captured substantial variation in the educational level of caregivers: about 8.0% of caregivers in the sample had no formal education; 13.3% had primary school education; 19.5% had their SSE/GCE; 14.2% had an Ordinary National Diploma (OND); 33.0% had Higher National Diploma (HND) or a bachelor’s (BSc) and 12.1% had postgraduate education. Less than half (44.8%) of caregivers were female. About 38.0% of caregivers were aged 18–29, while 18.5% were aged 40 or older. Most of the sample was from Abuja: 64.2% of caregivers were from Abuja, 32.7% were from Nasarawa and only 3.1% were from Adamawa. Most caregivers (59.0%) took care of adolescent girls aged 9–11, while 30.2% cared for adolescent girls aged 12–14.

**Table 1:** Caregiver Characteristics, Motivation and Ability, and their Relationship to HPV Vaccination.

Variables	(1) n (%) 4830 (100)	(2) Vaccinated n (%) 2604 (53.9)	P value
**Education of caregiver**			<0.001
No formal education	384 (8.0)	336 (87.5)	
Primary School Certificate	640 (13.3)	494 (77.2)	
SSE/GCE	942 (19.5)	518 (55.0)	
OND	686 (14.2)	322 (46.9)	
HND/BSc	1594 (33.0)	701 (44.0)	
Postgraduate degree	584 (12.1)	233 (39.9)	
**Gender of caregiver**			<0.001
Male	2674 (55.4)	1332 (49.8)	
Female	2156 (44.8)	1272 (59.0)	
**Age of caregiver**			<0.001
18–29	1834 (38.0)	1113 (60.7)	
30–39	2101 (43.5)	1105 (52.6)	
40 and older	895 (18.5)	386 (43.1)	
**State**			<0.001
Abuja	3099 (64.2)	1502 (48.5)	
Adamawa	152 (3.1)	43 (28.3)	
Nasarawa	1579 (32.7)	1059 (67.1)	
**Age of adolescent girl**			<0.001
9–11	2851 (59.0)	1737 (60.9)	
12–14	1459 (30.2)	726 (49.8)	
15–17	520 (10.8)	141 (27.1)	
**Motivation: Getting the girl vaccinated against HPV is important**			<0.001
All other responses	2337 (48.4)	1083 (46.3)	
Strongly agree	2493 (51.6)	1521 (61.0)	
**Ability: I know where to get the HPV vaccine**			<0.001
All other responses	3021 (62.5)	1305 (43.2)	
Strongly agree	1809 (37.5)	1299 (71.8)	
**Knowledge: Age at which vaccination is given**			<0.001
All other ages	2570 (53.2)	1062 (41.3)	
9–14 years	2260 (46.8)	1542 (68.2)	
**Exposure: Seen or heard ad about HPV vaccine in last 3 months**			<0.001
No	1552 (32.1)	296 (19.1)	
Yes	3278 (67.9)	2308 (70.4)	
**Girl in my care has received the HPV vaccine**			
No	2226 (46.1)	–	
Yes	2604 (53.9)	–	

Table 1, Column 1 also shows that about 51.6% of caregivers had high motivation, characterized by their strong agreement with the statement that HPV vaccination was important for their girl child. The level of ability was lower than the level of motivation, with about 37.5% of caregivers reflecting high ability through their strong agreement with the statement that they knew where to access the HPV vaccine. Knowledge of HPV vaccination was relatively low, with 46.8% correctly identifying ages 9–14 as the appropriate age for HPV vaccination. Exposure to HPV vaccine advertising in the prior three months was high: 67.9% of caregivers reported having seen or heard any advertisements or messages about HPV vaccination in the last 3 months. In terms of actual vaccine adoption, over half (53.9%) of caregivers reported that the adolescent girl in their care had received the HPV vaccine. As mentioned earlier, for caregivers of girls 9–14, this percentage was higher (57.2%).

### Bivariate analyses

Table 1, column 2 shows cross tabulations between socio-demographic characteristics of caregivers, motivation and ability, and HPV vaccination rates. The data shows a significant negative association between a caregiver’s education and HPV vaccination: HPV vaccination was nearly 48 percentage points higher among adolescents whose caregivers had no formal education compared to adolescents whose caregivershad postgraduate education (87.5% vs 39.9%). Adolescent girls’ vaccination rates were higher when caregivers were women compared to men (59.0% vs 49.8%). The vaccination rate was higher for adolescents with younger caregivers: 60.7% of caregivers aged 18–29 reported that their child was vaccinated compared to 43.1% of caregivers aged 40 and older.

Vaccine uptake was higher in Nasarawa (67.1%) than in Abuja (48.5%) or Adamawa (28.3%). Younger adolescents had higher vaccination rates than older adolescents: 60.9% of adolescent girls 9–11 were vaccinated compared to 49.8% of adolescent girls 12–14 and 27.1% of adolescent girls 15–17. High motivation was associated with a 15-percentage point higher rate of vaccination (61.0% versus 46.3%). High ability was associated with a nearly 29-percentage point higher vaccination rate: 71.8% of caregivers with high ability reported that their child was vaccinated compared to 43.2% of caregivers with lower ability. Knowledge of the age at which a girl should be vaccinated was associated with a nearly 27 percentage point higher vaccination rate (68.2% vs 41.3%). The variable measuring exposure to messages related to the HPV vaccine showed a 51 percentage point differential in the vaccination rate: 70.4% of caregivers exposed to HPV advertising messages reported that their child was vaccinated compared with 19.1% of caregivers who were not exposed to HPV vaccine messages.

Table 2 shows the relationships between education, demographic characteristics, motivation, ability and exposure to vaccine advertising. The data shows a negative association between caregivers’ education and exposure to HPV vaccine advertising. Caregivers with low levels of education had higher exposure to HPV vaccination messages than caregivers with high levels of education: more than 90% of caregivers with no formal education or only a primary school education had seen or heard ads about the HPV vaccine compared to just over 50% of caregivers with HND/BSc or postgraduate education. Female caregivers were more likely to be exposed to HPV vaccine ads than male caregivers (72.0% vs 64.5%). Younger caregivers were more likely to be exposed to vaccine advertising than older caregivers: 72.1% of caregivers aged 18–29 were exposed to HPV vaccine messages compared with 54.7% of caregivers aged 40 and older. Caregivers in Nasarawa (80.3%) were more likely to be exposed to HPV vaccine messages than caregivers in Abuja (62.5%) or Adamawa (28.3%). Caregivers of adolescents aged 9–11 were more likely to have been exposed to HPV vaccine messaging than caregivers of adolescents aged 15–17 (70.2% vs 47.3%).

**Table 2:** Caregiver Characteristics, Motivation and Ability, and Exposure to HPV Vaccine Advertising.

Variables	Exposed n (%) 3278 (67.9)	P value
**Education of caregiver**		<0.001
No formal education	366(95.3)	
Primary School Certificate	604 (94.4)	
SSE/GCE	692 (73.5)	
OND	446 (65.0)	
HND/BSc	857 (53.8)	
Postgraduate degree	313 (53.6)	
**Gender of caregiver**		<0.001
Male	1726 (64.5)	
Female	1552 (72.0)	
**Age of caregiver**		<0.001
18–29	1323 (72.1)	
30–39	1465 (69,7)	
40 and older	490 (54.7)	
**State**		<0.001
Abuja	1936 (62.5)	
Adamawa	74 (28.3)	
Nasarawa	1268 (80.3)	
**Age of adolescent girl**		<0.001
9–11	1737 (70.2)	
12–14	726 (70,7)	
15–17	141 (47.3)	
**Motivation: Getting the girl vaccinated against HPV is important**		0.004
All other responses	1539 (65.9)	
Strongly agree	1739 (69.8)	
**Ability: I know where to get the HPV vaccine**		<0.001
All other responses	1831 (60.6)	
Strongly agree	1447 (80,0)	
**Knowledge: Age at which vaccination is given**		<0.001
All other ages	1577 (61.4)	
9–14 years	1701 (75.2)	

Caregivers with high motivation were more likely to be exposed to vaccine advertising than caregivers with lower motivation (69.8% vs 65.9%), although the magnitude of the difference was relatively small. Caregivers with high ability were more likely be exposed to vaccine advertisements than caregivers with lower ability (80.0% vs 60.6%). Knowledge of the age at which a child should be vaccinated was associated with higher exposure to HPV vaccine advertising (75.2% vs 61.4%).

Previous social media research on HPV vaccination has found interactions between exposure to vaccine advertising and motivation and ability. Table 3 explores whether the relationships between education, demographic characteristics, motivation, ability and HPV vaccination vary by caregivers’ exposure to vaccine advertising.

**Table 3:** Caregiver Characteristics, Motivation, and Ability and HPV Vaccination, by Exposure to Vaccine Ads.

Variables	(1) Vaccinated (Exposed) n (%) 2308 (70.4)	P value	(2) Vaccinated (Not exposed) n (%) 296 (19.1)	P value
**Education of caregiver**		<0.001		0.422
No formal education	331 (90.4)		5 (27.5)	
Primary School Certificate	486 (80.5)		8 (22.2)	
SSE/GCE	480(69.4)		38 (15.2)	
OND	278 (62.3)		44 (18.3)	
HND/BSc	549 (64.1)		152 (20.6)	
Postgraduate degree	184 (58.8)		49 (18.1)	
**Gender of caregiver**		<0.001		0.367
Male	1158 (67.1)		5 (18.4)	
Female	1150 (74.1)		8 (20.2)	
**Age of caregiver**		<0.001		0.348
18–29	1015 (76.7)		98 (19.2)	
30–39	993 (67.8)		112 (17.6)	
40 and older	300 (61.2)		88 (21.2)	
**State**		<0.001		0.163
Abuja	1284 (66.3)		218 (18.7)	
Adamawa	33 (44.6)		10 (12.8)	
Nasarawa	991 (78.2)		68 (21.9)	
**Age of adolescent girl**		<0.001		0.025
9–11	1559 (78.0)		178 (20.9)	
12–14	645 (62.5)		81 (19.0)	
15–17	104 (42.3)		37(13.5)	
**Motivation: Getting the girl vaccinated against HPV is important**		<0.001		<0.001
All other responses	984 (63.9)		99 (12.4)	
Strongly agree	1324 (76.1)		197 (26.1)	
**Ability: I know where to get the HPV vaccine**		<0.001		<0.001
All other responses	1143 (62.4)		162 (13.6)	
Strongly agree	1165 (80.5)		134 (37.0)	
**Knowledge: Age at which vaccination is given**		<0.001		<0.001
All other ages	962 (61.0)		100 (10.1)	
9–14 years	1346 (79.1)		196 (35.1)	

Table 3 shows the bivariate relationship between socio-demographic characteristics and vaccine uptake among caregivers who were exposed to vaccine advertising (Column 1) and caregivers who were not (Column 2). Among caregivers exposed to HPV vaccine messaging, there was a negative relationship between education and vaccination: children of caregivers with postgraduate education had a 31 percentage points lower HPV vaccination rate than children of caregivers with no formal education (58.8% versus 90.4%). By contrast, there was no statistically significant relationship between education and HPV vaccination among caregivers who were not exposed to vaccine advertising.

Table 3, column 1 also shows that among caregiver exposed to vaccine advertising, women were more likely to get their child vaccinated than men (74.1% vs 67.1%). By contrast, column 2 shows that there was no gender-based differential in vaccine uptake among caregivers who were not exposed to HPV vaccine advertising. The relationship between the age of the caregiver and the child’s vaccination status is also moderated by exposure to HPV vaccine advertising: among caregivers exposed to HPV vaccine messages, 76.7% of 18–29-year-olds got their child vaccinated, compared to 61.2% of those 40 and older; among caregivers who were not exposed to vaccine advertising, the caregiver’s age is not associated with the child’s vaccination status. Exposure to HPV vaccine advertising also moderates the relationship between the state the caregiver lives in and vaccine uptake: among caregivers exposed to vaccine messages, the HPV vaccination rate is 12 percentage points (78.2% vs 66.3%) higher in Nasarawa than in Abuja – while there is no association between which state a caregiver lives in and HPV vaccination among caregivers not exposed to HPV vaccine advertising.

The associations between the caregiver’s motivation and vaccine uptake, the caregiver’s ability and vaccine uptake, and the caregiver’s knowledge and vaccine uptake are significant and in the same direction (i.e., greater motivation, ability and knowledge are associated with a higher vaccination rate) whether the caregiver is exposed to HPV vaccine messaging or not. However, the relationship between ability and HPV vaccination appears to be slightly stronger among caregivers who are not exposed to vaccine advertising: there is a 23.4 percentage point difference in HPV vaccination by the caregiver’s ability (37.0% vs 13.6%) among caregivers not exposed to vaccine advertising compared to an 18.1 percentage point difference by ability among caregivers who are exposed to vaccine advertising (80.5% versus 62.4%). Similarly, the magnitude of the differential in vaccination knowledge appears to be greater for caregivers who are not exposed to HPV vaccine advertising (35.1% vs 10.1% or 25.0 percentage points) than for caregivers who are exposed to advertising (79.1% vs 61.0% or 18.1 percentage points).

### Multivariate analysis

#### Main effects.

Table 4 presents adjusted odds ratios (aORs) of HPV vaccination among adolescent girls by caregiver’s education, demographic characteristics, motivation and ability. Column 1 shows the main effects, while column 2 shows interactions with advertising exposure. After adjusting for demographic characteristics, motivation and ability, caregivers with lower levels of education were more likely to get their girl child vaccinated: caregivers with no formal education had a 3 times high odds ratio of getting their child vaccinated, compared to caregivers with HND or higher education (aOR=3.01; 95% CI: 1.52–5.93). Caregivers with primary school education had more than twice the odds of getting their child vaccinated compared with caregivers who had HND or higher education (aOR=2.41; 95% CI: 2.02–2.87).

**Table 4:** Odds Ratios from Logistic Regression Associated with HPV Vaccination (n = 4830).

Variables	(1) Main Effects	P value	(2) Interactions Only*	P value
**Education of caregiver**				
No formal education	3.01 (1.52–5.93)	0.002		
Primary School Certificate	2.41 (2.02–2.87)	<0.001		
SSE/GCE	1.45 (0.84–2.50)	0.187		
OND	1.22 (0.81–1.83)	0.340		
HND/BSc/ Postgraduate degree	1.00			
No formal education x exposure			2.82 (1.24–6.41)	0.013
Primary School x exposure			2.54 (1.13–5.67)	0.024
SSE/GCE x exposure			2.67 (1.57–4.54)	<0.001
OND x exposure			1.10 (0.91–1.34)	0.317
**Gender of caregiver**				
Male	1.00			
Female	0.95 (0.80–1.12)	0.561		
Female x exposure			0.90 (0.83–0.97)	0.009
**Age of caregiver**				
18–29	1.00			
30–39	1.02 (0.87–1.19)	0.800		
40 and older	1.09 (1.01–1.17)	0.023		
30–39 x exposure			1.07 (0.99–1.14)	0.053
40 and older x exposure			0.79 (0.42–1.46)	0.454
**State**				
Abuja	1.00			
Adamawa	0.44 (0.43–0.44)	<0.001		
Nasarawa	1.60 (1.50–1.70)	<0.001		
Adamawa x exposure			0.58 (0.54–0.62)	<0.001
Nasarawa x exposure			1,11 (1.10–1.12)	<0.001
**Age of adolescent girl**				
9–11	2.73 (1.74–4.29)	<0.001		
12–14	2.11 (1.66–2.67)	<0.001		
15–17	1.00			
9–11 x exposure			2.76 (2.32–3.28)	<0.001
12–14 x exposure			1.66 (1.32–2.09)	<0.001
**Motivation: Getting the girl vaccinated against HPV is important**				
All other responses	1.00			
Strongly agree	1.29 (0.96–1.73)	0.095		
Strongly agree x exposure			0.65 (0.45–0.93)	0.020
**Ability: I know where to get the HPV vaccine**				
All other responses	1.00			
Strongly agree	2.05 (1.88–2.24)	<0.001		
Strongly agree x exposure			0.41 (0.37–0.45)	<0.001
**Knowledge: Age at which vaccination is given**				
All other ages	1.00			
9–14 years	2.28 (2.23–2.34)	<0.001		
9–14 x exposure			0.46 (0.40–0.52)	<0.001
**Exposure: Seen or heard ad about HPV vaccine in last 3 months**				
No	1.00			
Yes	6.87 (6.20–7.61)	<0.001		
Motivation*Ability*Exposure			2.30 (2.03–2.62)	<0.001
R-squared	27.0%		28.40	

*For ease of reading, main effects are not shown for this model.

The caregiver’s gender was not associated with vaccine uptake in the main effects model. Caregivers who were 40 and older were more likely to get their child vaccinated than caregivers who were 18–29 (aOR=1.01; 95% CI: 1.01–1.17). Caregivers in Nasarawa were more likely to get their girl child vaccinated than caregivers in Abuja (aOR=1.60; 95% CI: 1.50–1.70), while caregivers in Adamawa were less likely to get their girl child vaccinated than caregivers in Abuja (aOR=0.44; 95% CI: 0.43–0.44). Both caregivers of adolescent girls aged 9–11 (aOR=2.73; 95% CI: 1.74–4.29) and caregivers of adolescent girls aged 12–14 (aOR=2.11; 95% CI: 1.66–2.67) were more likely to get their child vaccinated than caregivers of adolescent girls aged 15–17.

After adjusting for other variables in the model, a caregiver’s motivation was not associated with vaccine uptake. However, the caregiver’s ability was associated with higher vaccine uptake: caregivers who strongly agreed they knew where to obtain the HPV vaccine had twice the odds ratio of getting their child vaccinated (aOR=2.05; 95% CI: 1.88–2.24). Knowing the age at which the child should be vaccinated was associated with a higher vaccination rate (aOR=2.28; 95% CI: 2.23–2.34). Exposure to HPV vaccine advertising had a powerful association with HPV vaccine uptake in the main effects model: caregivers who reported being exposed to HPV vaccine messaging had a nearly 7 times higher odds ratio of getting their child vaccinated (aOR=6.87; 95% CI: 6.20–7.61).

#### Interactions with exposure.

In line with previous social media research and with findings from the bivariate analysis shown in Table 3, we tested the presence of interactions in the multivariate model. We tested whether advertising exposure moderates the relationship between education, demographic characteristics, motivation and ability and vaccine uptake. Only interactions are shown in Column 2.

Column 2 of Table 4 shows that the negative relationship between education and vaccine uptake is stronger for caregivers who are exposed to HPV vaccine advertising: caregivers with no formal education have a higher likelihood of getting their child vaccinated than caregivers with HND or higher education when they are exposed to advertising. In other word the relationship between the education of caregiver and HPV vaccination varies by exposure to advertising. Similar interactions are observed between exposure to HPV vaccine messages and primary school education as well as SSE/GCE level education. Although the cross-sectional nature of this study does not allow us to make causal inferences, the findings are consistent with the hypothesis that caregivers with lower levels of education (none, primary or SSE/GCE) benefit to a significantly greater extent from HPV vaccine advertising than caregivers with higher levels of education (HND/BSc or postgraduate).

Column 2 also shows an interaction between gender and exposure to HPV vaccine advertising. Women exposed to advertising are less likely to get their child vaccinated than men exposed to advertising. These findings support the hypothesis that male caregivers are more able to take advantage of HPV vaccine advertising than female caregivers.

Advertising exposure also moderates the effect of region on vaccine uptake. Caregivers who are exposed to vaccine advertising in Adamawa are less likely to get their child vaccinated than caregivers who are exposed to vaccine advertising in Abuja. This finding suggests the hypothesis that caregivers in Abuja are more able to translate exposure to HPV vaccine advertising into getting their child vaccinated. Caregivers in Nasarawa who are exposed to advertising are more likely to get their child vaccinated against HPV than caregivers in Abuja who are exposed to vaccine advertising. This suggests the hypothesis that HPV vaccine advertising successfully translates into HPV vaccine uptake in Nasarawa compared to Abuja.

Relative to caregivers who are not exposed to advertising, caregivers of adolescent girls aged 9–11 and caregivers of adolescent girls aged 12–14 who are exposed to advertising are more likely than caregivers of adolescent girls 15–17 who are exposed to advertising to get their child vaccinated. This suggests the hypothesis that HPV vaccine advertising translates more successfully into HPV vaccine uptake for caregivers with children aged 9–11 and 12–14 than for caregivers with adolescents 15–17.

The data also shows interactions between advertising exposure and motivation, ability and knowledge variables. The data suggest that the positive relationship between motivation and vaccine uptake is stronger for caregivers who are not exposed to HPV vaccine advertising. In other words, motivation appears to be a more important driver of vaccine uptake among caregivers who are not exposed to HPV vaccine advertising. This finding is counterintuitive. Similar findings are observed for ability and knowledge of the correct age at which to vaccinate the girl child. The caregiver’s ability to get the child vaccinated appears to matter more when the caregiver is not exposed to HPV vaccine messaging than when they are. Similarly, knowledge of the correct age of vaccination is more strongly associated with higher HPV vaccination when the caregiver is not exposed to the campaign. These findings are also counterintuitive. Given the cross-sectional nature of this data, these findings must be interpreted with caution.

Finally, we tested for an interaction between motivation, ability, and exposure to vaccine advertising. The data show that the being exposed to HPV vaccine advertising is particularly important for caregivers who have high motivation and high ability. The Fogg Behavior Model states that behavior happens when motivation, ability and a prompt happen in the same moment. Our data demonstrates the interaction between motivation, ability and prompt implied by the Fogg Behavior Model. There is a multiplicative effect of exposure to HPV vaccine advertising among caregivers who are motivated to get their child vaccinated and have the ability to do so.

## Discussion

This study provides critical insights into the complex dynamics influencing HPV vaccine uptake among adolescent girls whose caregivers are social media users. By leveraging data from a digitally recruited sample of caregivers, we investigated the interplay between education, motivation, ability, and exposure to vaccine advertising. Our findings highlight several key trends: caregivers with lower education levels were more likely to vaccinate their children. This relationship was moderated by exposure to vaccine advertising, as children of less educated caregivers exposed to messages promoting the HPV vaccine showed higher vaccine uptake. Motivation and ability were positively associated with vaccine uptake, but their influence was stronger among caregivers not exposed to HPV vaccine messaging. These results underscore the nuanced roles of education, motivation, ability, and digital engagement in shaping health behaviors, particularly in the context of vaccine hesitancy.

### Education and vaccine uptake: a complex relationship

Contrary to the assumption that higher education universally promotes health-seeking behaviors, our study aligns with prior findings from Kenya and Nigeria that suggest a paradoxical relationship between education and vaccine hesitancy [4–5]. Tertiary-educated caregivers showed lower HPV vaccine uptake, possibly due to critical evaluations of campaigns or exposure to misinformation. This underscores the need for targeted communication strategies that address the informational preferences and skepticism of highly educated populations. It is important to understand what types of messages are considered trustworthy by more educated Nigerians. Alternatively, it may be important to identify trusted sources of information, other than social media, to reach more educated respondents effectively. Higher education might lead to increased access to conflicting sources of information, including anti-vaccine narratives, which resonate more strongly with individuals who critically assess messages on social media platforms. Addressing this requires tailored messages developed in partnership with trusted leaders, such as health professionals or educators, to build credibility among educated caregivers.

Qualitative insights from similar contexts suggest that misinformation about vaccine safety or effectiveness may be particularly persuasive among tertiary-educated individuals. For example, rumors linking vaccines to infertility or promoting “natural immunity” may resonate differently depending on the recipient’s education level and media consumption patterns [17]. Integrating targeted, evidence-based interventions into professional forums, associations or alumni networks may be an effective strategy for reaching this demographic.

Similar findings have been observed globally, where HPV vaccination efforts relying solely on mass communication faced challenges reaching more highly educated groups. For instance, a recent study from Kenya emphasized that addressing vaccine hesitancy among educated caregivers required integrating tailored, evidence-based content delivered through trusted community channels beyond digital media alone [5]. The study also suggested that differences in information access — including greater exposure to digital or online misinformation — may partly explain why more educated and urban caregivers demonstrated higher levels of hesitancy. Likewise, a recent study [16] highlighted that while socioeconomic disparities in vaccine uptake are pervasive, the mechanisms—such as trust in information sources and susceptibility to misinformation—vary across education levels and contexts, reinforcing the need for differentiated communication strategies. These insights align with our findings and suggest that optimizing HPV vaccination campaigns in Nigeria will require multi-modal outreach efforts that balance digital targeting with community-based interventions.

### The role of HPV vaccine advertising

Exposure to vaccine advertising emerged as powerfully associated with HPV vaccination. Caregivers exposed to HPV messaging were significantly more likely to vaccinate their children, with an almost sevenfold increase in the odds ratio. However, our findings also reveal disparities in exposure, with caregivers with higher education less likely to recall HPV vaccine messages. With social media being the largest source of HPV vaccine messaging in Nigeria [12], this highlights the dual role of media as both a vector for misinformation and an effective platform for health promotion – as algorithms create echo chambers encouraging vaccination among less educated caregivers, while isolating more educated ones from campaign messages.

Social media algorithms, which often reinforce existing beliefs, may exacerbate these disparities. Less educated caregivers may be more exposed to localized or emotional appeals, while educated caregivers may encounter content that aligns with their critical assessments, including anti-vaccine sentiment. Adjusting algorithmic targeting to ensure diverse messaging reaches all audiences is crucial. Additionally, partnerships with social media platforms could explore “myth-busting” campaigns where common misinformation is addressed directly within users’ feeds.

### Motivation, ability, and the importance of prompts

The Fogg Behavior Model provides a useful lens for interpreting our findings. While both motivation and ability were associated with vaccine uptake, their effects were more pronounced among caregivers who lacked exposure to HPV messaging. This suggests that, in the absence of a strong external prompt (e.g., an advertisement), caregivers’ motivation and ability become even more important as drivers of adolescents’ vaccination. The data also reveals an interaction between motivation, ability and exposure to advertising. The Fogg Behavior Model posits that behavior is most likely to happen when all 3 elements are present. The interaction between motivation, ability, and prompts underscores the importance of designing campaigns that simultaneously address all three factors. This is one of the first studies to provide data supporting the interaction between motivation, ability and prompt and highlights the traction gained by targeting caregivers who are motivated and able to adopt a behavior.

### Regional and demographic variations

Geographic and demographic factors also shaped HPV vaccination rates. Caregivers in Nasarawa demonstrated higher vaccine uptake than those in Abuja or Adamawa, reflecting regional differences in campaign implementation or access to vaccination sites.

The stronger effect of advertising in Nasarawa may indicate that regional outreach strategies were successfully tailored to the local population’s preferences and barriers. Conversely, the weaker outcomes in Adamawa suggest the need for more granular exploration of health system access issues or localized myths about vaccines that may impede uptake. Incorporating community leaders or engaging local influencers could help bridge these gaps.

Caregivers of younger adolescents were more likely to vaccinate their children, emphasizing the importance of tailoring interventions to demographic subgroups. The findings also suggest that caregivers of adolescents ages 9–14 may already perceive vaccination as timely and relevant. Conversely, strategies to engage caregivers of older adolescents might include messaging focused on the benefits of vaccination even after the ideal window.

Gender differences were less pronounced overall, though female caregivers responded differently to advertising prompts compared to their male counterparts, indicating potential gender-specific barriers or facilitators to vaccination.

### Implications for policy and practice

Our findings offer several actionable recommendations for improving HPV vaccine uptake in Nigeria and similar contexts:

1. **Develop Nuanced Messaging for Educated Caregivers:** Craft campaigns addressing skepticism and critical evaluations, with a focus on behavioral insights-informed messaging delivered by trusted sources.
2. **Enhance Advertising Reach:** Address disparities in message exposure by diversifying digital targeting strategies and incorporating other channels to reach underexposed groups.
3. **Enhance Social Media Targeting**: Partner with social media platforms to refine algorithms, ensuring balanced exposure to vaccine information across education levels.
4. **Develop Prompts that Motivate Caregivers:** In the Fogg Behavior Model (FBM), a spark is a specific type of prompt that is designed to motivate action by eliciting an emotional response. Unlike other types of prompts that rely on existing motivation or focus on simplifying tasks (ability-focused prompts), a spark aims to increase motivation directly to encourage the desired behavior. Sparks tap into emotions such as fear, hope, excitement, or a sense of urgency to drive motivation. For example, a health campaign might use emotional stories of cervical cancer survivors to spark motivation for HPV vaccination.
5. **Prioritize Regional Customization**: Focus on addressing unique barriers in regions like Adamawa while amplifying successful strategies in Nasarawa.

### Study limitations and future research

This study’s reliance on self-reported data may introduce recall or social desirability bias regarding vaccination status. Additionally, the cross-sectional design precludes causal inference, limiting our ability to determine the directionality of observed relationships. A longitudinal approach would have been stronger for this study but was not feasible withing the resources available for the study. Future research should consider longitudinal study designs to capture temporal changes in vaccine-related behaviors and explore alternative recruitment methods to improve representativeness. The digital recruitment strategy also overrepresents younger, urban, and digitally connected caregivers, which may not reflect the broader population of caregivers in Nigeria.

## Conclusion

This study highlights the complexity of HPV vaccine uptake in Nigeria, highlighting how education, media exposure, and behavior intersect. By addressing disparities in vaccine messaging and tailoring strategies to demographic and regional contexts, public health interventions can better navigate the challenges of vaccine hesitancy and misinformation. These findings offer valuable insights for advancing HPV vaccination efforts in LMICs, ultimately contributing to the reduction of cervical cancer disparities globally.

## Supporting information

S1 Checklist Inclusivity in global research. (PDF)
